# A LATENT CLASS ANALYSIS OF INSTRUMENTAL ACTIVITIES OF DAILY LIVING (IADL) CAREGIVING NEEDS

**DOI:** 10.1093/geroni/igae098.4099

**Published:** 2024-12-31

**Authors:** Suyoung Kim, Sunshine Rote, Phillip Cantu, Flávia Andrade, Jacqueline Angel

**Affiliations:** University of Texas at Austin, Austin, Texas, United States; University of Louisville, Louisville, Kentucky, United States; University of Texas Medical Branch, Galveston, Texas, United States; University of Illinois at Urbana Champaign, Urbana, Illinois, United States; University of Texas at Austin, Austin, Texas, United States

## Abstract

**Objectives:**

Mexican American caregivers experience time-intensive caregiving situations yet studies on heterogeneity in caregiving needs are limited. This study (1) utilizes latent class analysis (LCA) to classify caregivers into groups based on care recipient IADL intensity and type of support need and (2) examines caregiver psychological outcomes by care recipient support need.

**Methods:**

Data from the Hispanic Established Populations for the Epidemiologic Study of the Elderly (Wave 7, N = 885) was analyzed using LCA. Characteristics of both caregivers and care recipients were included as predictors for a dyadic analysis, and caregiver perceived stress, depression, overall life satisfaction were investigated as tertiary outcomes.

**Results:**

Four distinct groups of Mexican American caregivers were identified by care recipient IADL assistance need: comprehensive (39%), social task only (29%), minimal (23%), and physical mobility only (9%). Care recipient cognitive functioning and nativity status as well as caregiver homeownership status were associated with IADL assistance need. Caregiver perceived stress was highest for comprehensive IADL assistance need and lowest for social task only need. Caregiver life satisfaction peaked for those providing care for care recipients with minimal need, and caregiver depressive symptoms were more prevalent for comprehensive and physical mobility only IADL assistance need.

**Discussion:**

Caregiving demands, especially for mobility and comprehensive social, household, mobility, and financial care needs, are associated with greater caregiver psychological burden. Targeted, culturally competent interventions aimed at improving the mental health of Mexican American caregivers should address the unique challenges with mobility and comprehensive care needs.

